# PPARγ Acetylation Orchestrates Adipose Plasticity and Metabolic Rhythms

**DOI:** 10.1002/advs.202204190

**Published:** 2022-11-17

**Authors:** Ying He, Alana B'nai Taub, Lexiang Yu, Yifan Yao, Ruotong Zhang, Tarik Zahr, Nicole Aaron, Joseph LeSauter, Lihong Fan, Longhua Liu, Ruya Tazebay, Jianwen Que, Utpal Pajvani, Liheng Wang, Rae Silver, Li Qiang

**Affiliations:** ^1^ Naomi Berrie Diabetes Center, Columbia University New York NY 10032 USA; ^2^ Department of Pathology and Cell Biology Columbia University New York NY 10032 USA; ^3^ Department of Psychology Columbia University New York NY 10027 USA; ^4^ Department of Neuroscience Barnard College New York NY 10027 USA; ^5^ Department of Molecular Pharmacology and Therapeutics Columbia University New York NY 10032 USA; ^6^ Department of Medicine Columbia University New York NY 10032 USA; ^7^ The Diabetes Obesity and Metabolism Institute The Icahn School of Medicine at Mount Sinai New York NY 10029 USA

**Keywords:** adipose plasticity, adipsin, BMAL1, metabolic rhythm, PPARγ acetylation

## Abstract

Systemic glucose metabolism and insulin activity oscillate in response to diurnal rhythms and nutrient availability with the necessary involvement of adipose tissue to maintain metabolic homeostasis. However, the adipose‐intrinsic regulatory mechanism remains elusive. Here, the dynamics of PPARγ acetylation in adipose tissue are shown to orchestrate metabolic oscillation in daily rhythms. Acetylation of PPARγ displays a diurnal rhythm in young healthy mice, with the peak at zeitgeber time 0 (ZT0) and the trough at ZT18. This rhythmic pattern is deranged in pathological conditions such as obesity, aging, and circadian disruption. The adipocyte‐specific acetylation‐mimetic mutation of PPARγ K293Q (aKQ) restrains adipose plasticity during calorie restriction and diet‐induced obesity, associated with proteolysis of a core circadian component BMAL1. Consistently, the rhythmicity in glucose tolerance and insulin sensitivity is altered in aKQ and the complementary PPARγ deacetylation‐mimetic K268R/K293R (2KR) mouse models. Furthermore, the PPARγ acetylation‐sensitive downstream target adipsin is revealed as a novel diurnal factor that destabilizes BMAL1 and mediates metabolic rhythms. These findings collectively signify that PPARγ acetylation is a hinge connecting adipose plasticity and metabolic rhythms, the two determinants of metabolic health.

## Graphical abstract

The acetylation of PPAR*γ* is dynamically regulated in diurnal rhythms to orchestrate metabolic oscillation and adipose plasticity. The PPARγ acetylation‐responsive downstream target adipsin is identified as a novel diurnal factor to regulate metabolic rhythm through destabilizing BMAL1 in an adipocyte‐intrinsic manner.

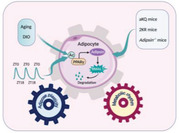

## 1 Introduction

All living organisms must maintain metabolic homeostasis to cope with nutrition fluctuations, diurnal rhythms, and acute stress. Metabolic rhythms are impacted by daily cycles of food intake and oscillations in metabolism, particularly in glucose metabolism and insulin sensitivity, two fundamental physiological activities. A growing body of evidence shows that circadian dysfunction is associated with metabolic dysregulation in aging,^[^ ^1^ ^]^ obesity,^[^ ^2^ ^]^ insulin resistance,^[^ ^3^ ^]^ type 2 diabetes,^[^ ^4^ ^]^ and cardiometabolic disease.^[^ ^5^ ^]^ Unfortunately, the modern human lifestyle challenges the maintenance of metabolic homeostasis. For example, frequent calorie‐dense food intake and continuous satiety diminish metabolic oscillation. This is particularly true for shiftworkers, who reportedly display decreased insulin sensitivity and increased risks of obesity and diabetes because of their disrupted circadian cycle.^[^ ^6^ ^]^ Hence, promoting metabolic rhythmicity is vital for maintaining metabolic health and curbing the current epidemic of metabolic diseases.^[^ ^7^ ^]^ The central nervous system has been demonstrated to play a critical role in controlling metabolic rhythm.^[^ ^3^, ^8^ ^]^ However, the mechanism by which metabolic rhythm is regulated peripherally and how it is impaired under pathophysiological conditions are not as well understood.

Adipose tissue is the primary energy storage organ, buffering nutrient fluctuations through lipid storage and release.^[^ ^9^ ^]^ Therefore, the metabolic plasticity of adipose tissue is essentially integrated into the systemic metabolic oscillation, but the key component linking adipose tissue with metabolic oscillation remains unknown. Peroxisome proliferator‐activated receptor gamma (PPARγ) is the dominant regulator of adipose biology in lipid synthesis and utilization, glucose uptake, hormonal response, adipokine production, and so on.^[^ ^10^ ^]^ Its thiazolidinedione (TZD) agonists work robustly in promoting insulin sensitivity and modulating glucose and lipid metabolism.^[^ ^11^ ^]^ In fact, PPARγ directly regulates circadian genes to reprogram the circadian clock in the liver upon nutritional challenges ^[^ ^12^ ^]^ and mediates the downregulation of BMAL1 in the adipose tissue of obese mice and humans.^[^ ^13^ ^]^ Thus, it is conceivable that PPAR*γ* may play a role in metabolic oscillation from both the lens of adipose plasticity and metabolic rhythm.

PPARγ is activated upon ligand binding to heterodimerize with RXR*α* and bind to DNA.^[^ ^14^ ^]^ The endogenous lipid ligands of PPARγ and their derivatives are believed to fluctuate in response to nutrient availability, leading to variation in PPARγ activity.^[^ ^15^ ^]^ The fluctuation of PPARγ activity can also be achieved at the transcriptional level as the expression of PPARγ is induced in adipose tissue by food intake to facilitate energy storage.^[^ ^16^ ^]^ In recent research, post‐translational modifications (PTMs) have been established as a new layer to regulate PPARγ function.^[^ ^17^ ^]^ In particular, deacetylation of PPAR*γ* by Sirtuin 1 (SirT1) at the Lys268 and Lys293 residues promotes brown remodeling of white adipose tissue (WAT),^[^ ^18^ ^]^ increases energy expenditure, and inhibits adiposity.^[^ ^19^ ^]^ Intriguingly, constitutively deacetylated PPARγ mimetic mutant K268R/K293R, namely 2KR, uncouples the significant adverse effects of TZD treatment (e.g., bone loss and cardiac hypertrophy) from the metabolic improvements and atheroprotective effect.^[^ ^19^, ^20^, ^21^ ^]^ This is likely achieved through the selective regulation of downstream target genes. As PTMs take place on the existing protein without the need for de novo synthesis, they may serve as an efficient way to modulate PPARγ activity and are ideally poised to link fluctuations in nutritional status, ligand availability, and signaling pathways with metabolic oscillation.

In consolidating the necessity of adipose tissue in metabolic homeostasis, the central role of PPARγ in adipocyte function, and the sensitive nature and importance of PTMs, we hypothesized that the acetylation of PPARγ is dynamically regulated in adipose tissue to affect metabolic rhythm. To test it, we investigated alterations in metabolic response and rhythm by PPARγ acetylation by employing a novel adipocyte‐specific PPARγ acetylation‐mimetic model (aKQ mice) in combination with the complementary deacetylation‐mimetic 2KR mouse model. We further revealed adipsin as a novel diurnal factor that destabilizes BMAL1 and regulates metabolic rhythms. Collectively, these data demonstrate that PPARγ acetylation provides a new bridge to connect metabolic oscillations and diurnal rhythms, highlighting the possibility of intervening in the acetylation state of PPARγ to promote metabolic rhythmicity.

## 2 Results

### 2.1 PPARγ Acetylation is Dynamically Regulated in Metabolic Changes

**Figure 1:**
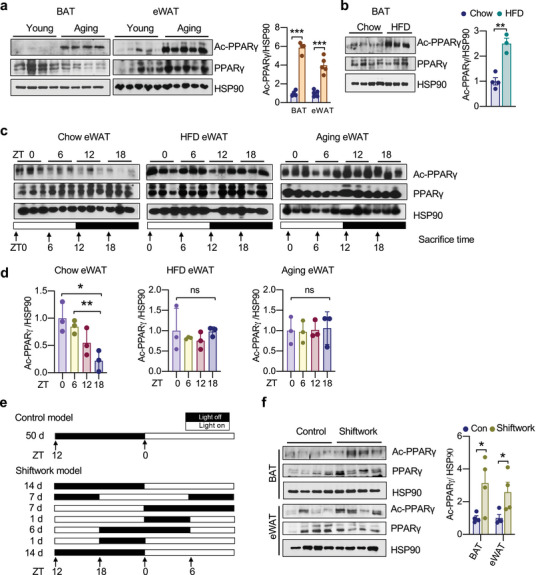
PPAR*γ* acetylation is dynamically regulated in metabolic changes. a) Western blot (WB) analysis and quantification of PPAR*γ* acetylation levels in BAT (n = 4, 4) and eWAT (n = 5, 5) in young (8 weeks) and aging (40 weeks) mice. b) WB analysis and quantification of PPAR*γ* acetylation levels in BAT of chow‐fed and DIO mice (n = 4,3). c,d) WB analyses and quantifications of PPAR*γ* acetylation levels in the eWAT of chow‐fed, DIO, and aging (1‐year‐old) mice sacrificed at 6 h intervals throughout the day (n = 3). e) Schematic design for shiftwork mouse model. f) WB analysis and quantification of PPAR*γ* acetylation levels in BAT (n = 4, 4) and eWAT (n = 4, 4) of shiftwork mice and control mice. Data are presented as mean ± SEM, two‐tailed Student's t‐test. *p < 0.05, **p < 0.01, and ***p < 0.001.

Aging and obesity are two primary etiologies for insulin resistance, often coinciding with the functional decline of adipose tissue. PPARγ acetylation levels were increased in the aged mice across different fat depots, including brown adipose tissue (BAT), epididymal WAT (eWAT), and inguinal WAT (iWAT) (**Figure** **1**a; Figure S1a, Supporting Information). In the parallel diet‐induced obese (DIO) mice, PPAR*γ* acetylation levels were similarly increased in the BAT as compared to chow‐fed controls (Figure 1b). These observations raise the possibility that PPARγ acetylation displays rhythmicity during the diurnal cycle in association with metabolic oscillation. In chow‐fed mice, PPARγ acetylation in eWAT oscillated with a daily rhythm, peaking at zeitgeber time 0 (ZT0; where ZT0 is the time of lights on; ZT12: lights off) and reaching a trough at ZT18 (Figure 1c,d). A similar pattern was seen in BAT (Figure S1b,c, Supporting Information). Notably, this oscillation of PPARγ acetylation was blunted by both HFD feeding and aging (Figure 1c,d; Figure S1b,c, Supporting Information). Together, these data indicate that PPARγ acetylation oscillates with diurnal rhythms and is positively associated with metabolic derangements.

Disruption of circadian rhythm leads to metabolic dysfunctions associated with aging,^[^ ^22^ ^]^ obesity,^[^ ^23^ ^]^ adipocyte hypertrophy, and compromised adipose tissue integrity.^[^ ^24^ ^]^ To explore the possibility that circadian disruption impacts PPARγ acetylation, we adopted a shiftwork mouse model ^[^ ^25^ ^]^ (Figure 1e). There were consistent increases in PPARγ acetylation in both BAT and WAT by shiftwork (Figure 1f). Intriguingly, the distinct functions of WAT and BAT were both impaired with the concurrent increase of PPARγ acetylation. In the lipid‐storing eWAT, the adipogenic markers and lipid synthetic genes were repressed by shiftwork (Figure S1d, Supporting Information). In contrast, the lipid‐consuming BAT showed upregulation of lipogenic genes accompanied by enlarged lipid droplets (Figure S1e,f, Supporting Information). These findings highlight the fact that acetylation is a key PTM of PPARγ associated with both metabolic impairment and diurnal rhythm disruption.

### 2.2 Generation of Adipocyte‐Conditional PPARγ Acetylation‐Mimetic Mouse Model

**Figure 2:**
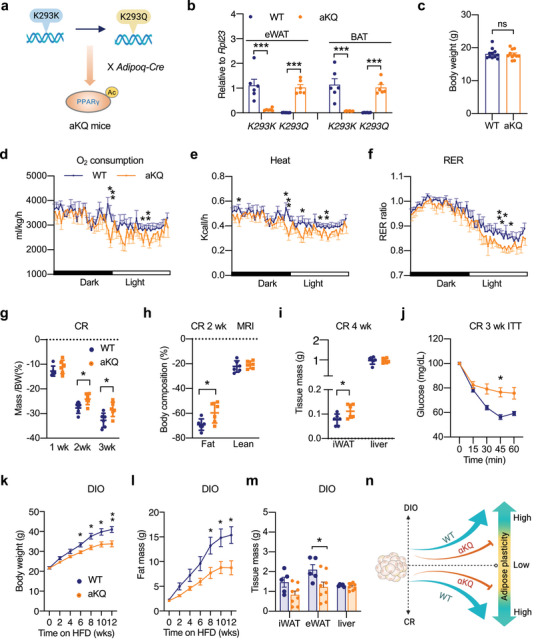
Constitutive PPARγ acetylation in adipocytes impairs adipose plasticity. a) Schematic outline of aKQ mouse breeding strategy. b) Gene expression validation by qPCR in eWAT and BAT from WT and aKQ mice (n = 6, 6). Ribosomal Protein L23 (Rpl23) was used as the reference gene. c) Body weight of male WT and aKQ mice on chow diet feeding at ≈10 weeks old (n = 11, 11). d–f) Indirect calorimetric analyses of 14‐weeks‐old male WT and aKQ mice fed a chow diet at room temperature (n = 9, 6); d) Oxygen consumption (normalized to body weight); e) Heat production (normalized to body weight); f) RER. *p < 0.05 at multiple detection points. g–j) Male WT and aKQ mice subjected to calorie restriction (CR) (n = 6, 6); g) body weight changes during the first 3 weeks CR; h) body composition at 2 weeks of CR; i) sacrificed tissue mass at 4 weeks of CR; j) ITT after 3 weeks of CR. k–m) 4‐weeks‐old male WT and aKQ mice on HFD for 12 weeks (n = 5, 8); k) body weight curve; l) fat mass curve on HFD feeding; m) tissue weight at sacrifice. n) Schematic diagram of impaired adipose plasticity in aKQ mice. The schemes in (a) and (n) were created using BioRender. Data are presented as mean ± SEM, *p < 0.05, **p < 0.01 by two‐tailed Student's t‐test.

PPARγ deacetylation promotes energy expenditure to favor a lean phenotype, as shown by the whole‐body knock‐in of deacetylation‐mimetic 2KR mutant.^[^ ^19^ ^]^ To determine whether PPARγ acetylation in adipose tissue accounts for this metabolic function, we generated a conditional PPARγ mutant knock‐in model (aKQ mice) by replacing Lys293 in exon 6 with glutamine to mimic constitutive acetylation (K293Q) and further breeding with an adipocyte‐specific Cre line (*Adipoq‐Cre*) (**Figure** **2**a; Figure S2a–c, Supporting Information). This strategy resulted in an efficient replacement of wildtype PPARγ in both WAT and BAT with K293Q mutant (Figure 2b), a complementary mutation to 2KR with minimal manipulation on endogenous PPARγ.^[^ ^18^ ^]^

The aKQ mice displayed normal development and body weight compared to wildtype (WT) control littermates on chow diet feeding at a young age (Figure 2c). However, indirect calorimetry analysis revealed that they exhibited modest decreases in oxygen consumption, heat production, and Respiratory Exchange Ratio (RER) (Figure 2) despite the remained diurnal rhythm (Figure S3a,b, Supporting Information), whereas their food intake and locomotor activity were not significantly affected (Figure S3c,d, Supporting Information). This decrease in energy expenditure eventually led to an obesity phenotype in aging aKQ mice (Figure S3e,f, Supporting Information). These findings reinforce the catabolic function of PPARγ deacetylation and specify it to a single residue K293 in adipocytes.

### 2.3 Constitutive PPARγ Acetylation Impairs Adipose Plasticity During Metabolic Adaptions

Adipocytes must efficiently adapt to metabolic changes, whereas diminished metabolic adaption, or impaired adipose plasticity, leads to metabolic dysregulations.^[^ ^26^ ^]^ We then asked whether constitutive PPARγ acetylation (aKQ mutation) would affect response to nutritional manipulations. Calorie restriction (CR) is well known for ameliorating metabolic detriments in aging and obesity.^[^ ^27^ ^]^ It also potently induces SirT1, the deacetylase of PPARγ.^[^ ^18^ ^]^ We then reasoned that the function of PPARγ acetylation might be exaggerated during CR. Indeed, the CR‐induced weight loss was attenuated in aKQ mice (Figure 2g), underlain by their better preservation of fat mass (Figure 2h) without affecting PPARγ expression in adipose tissues (Figure S3g,h, Supporting Information), resulting in a larger iWAT depot even after 4 weeks of CR (Figure 2i). One of the most profound effects of CR is improving insulin sensitivity; however, this improvement was diminished in aKQ mice (Figure 2j) despite their extreme glucose tolerance (Figure S3i, Supporting Information).

Adipose plasticity involves both reduction and expansion. Given the restrained fat shrinkage in aKQ mice, we asked whether this impaired adipose plasticity also leads to fat expansion. To test it, we adopted the DIO model. When starting high‐fat diet (HFD) at 4 weeks old, aKQ mice gained less weight than the control littermates; this was due exclusively to less fat mass gain rather than altering lean mass (Figure 2k,l; Figure S4a–c, Supporting Information). The eWAT depot sizes of aKQ mice were reduced by 30% (Figure 2m). Consistent with the CR model, PPARγ expression in aKQ mice remained unchanged at both the protein and gene expression levels in adipose tissues (Figure S4d,e, Supporting Information). Interestingly, this anti‐obesity phenotype disappeared in older aKQ mice (Figure S4f, Supporting Information). Taken together, constitutive acetylation of PPARγ in adipocytes impedes the response of adipose tissue to both reduction and expansion, indicating impaired adipose plasticity (Figure 2n).

### 2.4 PPARγ Acetylation Impairs Adipogenesis Accompanied by the Destabilization of BMAL1

**Figure 3:**
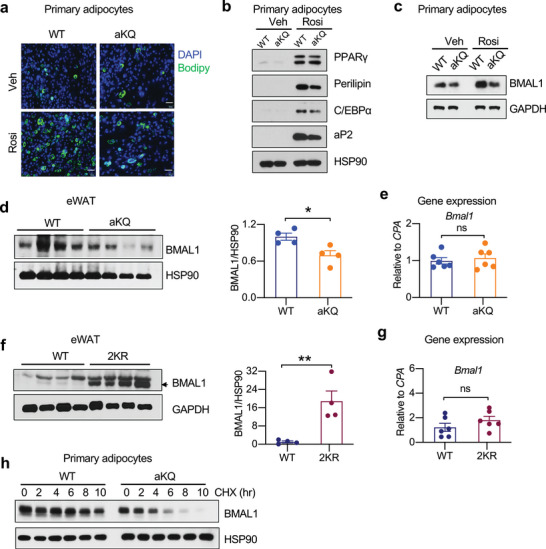
PPARγ acetylation destabilizes BMAL1 to inhibit adipogenesis. a–c) WT and aKQ primary adipocytes on day 8 of differentiation by vehicle (Veh) or rosiglitazone (Rosi) treatment; a) BODIPY staining showing lipid accumulation; Scale bar, 50 𝜇m. b,c) WB of adipocyte markers and BMAL1 in WT and aKQ adipocytes. d,e) WB of BMAL1 protein (n = 4, 4) and *Bmal1* gene expression by qPCR (n = 6, 6) in the eWAT of aKQ mice. *Cyclophilin A (CPA)* was used as the reference gene in qPCR analysis. f,g) WB of BMAL1 protein (n = 4, 4) and qPCR analysis of *Bmal1* gene expression (n = 6, 6) in the eWAT of WT and 2KR mice. *Cyclophilin A (CPA)* was used as the reference gene in qPCR analysis. h) BMAL1 protein in the aKQ primary adipocytes after treatment with cycloheximide (CHX) time course. Data are presented as mean ± SEM, *p < 0.05 and **p < 0.01 by two‐tailed Student's t‐test.

The decreased adipose tissue expansion in aKQ mice is underpinned by their impaired adipogenesis, as shown by the inhibited lipid accumulation and diminished adipocyte markers such as Perilipin, C/EBP*α*, and aP2 in their primary adipocytes (**Figure** **3**a,b). PPARγ agonist rosiglitazone (Rosi) treatment induced the expression of the K293Q mutant, validating its Adipoq‐Cre‐driven replacement of the WT allele (K293K) (Figure S5a, Supporting Information). The impaired adipogenesis by the K293Q mutation was reproduced in an engineered PPARγ knockout mouse embryonic fibroblast (MEF) cell line (*Pparg^−/‐^* KQ) with the doxycycline‐inducible reconstitution of PPAR*γ*2 (Figure S5b,c, Supporting Information) indicated by repressed adipocyte markers (Figure S5c,d, Supporting Information).

The core component of circadian regulation BMAL1 is well established as a pro‐adipogenic factor ^[^ ^28^ ^]^ and has been implicated in fat expansion and lipid metabolism.^[^ ^29^ ^]^ While exploring the impaired adipogenesis by KQ mutant, we observed an inhibited induction of BMAL1 in the aKQ adipocytes (Figure 3c). The decrease of BMAL1 protein levels was confirmed in vivo in the eWAT of aKQ mice; however, there were no significant changes in gene expression of *Bmal1* (Figure 3d,e). Consistently, in the eWAT of deacetylation‐mimetic 2KR mice, BMAL1 protein was increased but without changing gene expression (Figure 3f,g). This decoupled regulation of protein and mRNA levels suggests that the protein stability of BMAL1 is reduced by PPARγ acetylation. Further implicating adipocyte‐intrinsic regulation, BMAL1 in aKQ primary adipocytes was less stable than in WT cells (Figure 3h), an effect also seen in the reconstituted *Pparg^−/−^* KQ adipocytes (Figure S5e, Supporting Information). Moreover, we synchronized the circadian cycle with horse serum treatment in *Pparg^−/−^* WT and KQ adipocytes and observed overall repression of BMAL1 protein in the *Pparg^−/−^* KQ cells despite their increased expression of *Bmal1* (Figure S5f,g, Supporting Information). We conclude that PPARγ acetylation derails BMAL1 protein levels, thus impeding adipogenesis and possibly affecting metabolic rhythm.

### 2.5 Acetylation of PPARγ in Adipocytes Regulates Metabolic Oscillation in the Diurnal Cycle

**Figure 4:**
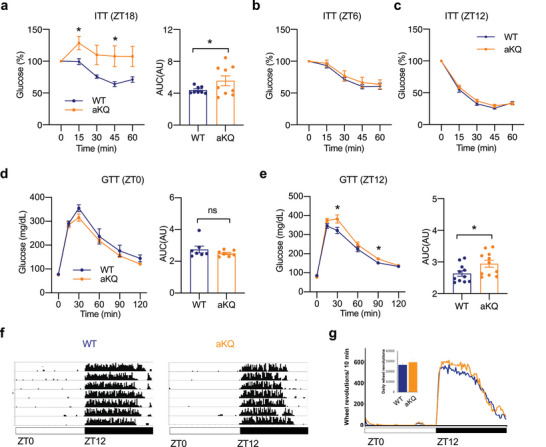
Altered metabolic rhythm in aKQ mice. Metabolic characterizations of male WT and aKQ mice on chow diet feeding at approximately 10‐weeks‐old; a–c) ITT and AUC at ZT18 (n = 8, 9), ZT6 (n = 6, 6), and ZT12 (n = 11, 11), respectively; d,e) GTT and associated AUC at ZT0 (n = 7, 7) and ZT 12 (n = 11, 11). f) Actograms showing the daily wheel‐running activity of two representative animals (WT and aKQ). The 12:12 lighting cycle is shown by the horizontal bar on the bottom; white denotes light, and black denotes darkness. The amount of running is represented by the black shading on each consecutive day for 7 days. g) The line graph shows the average daily profile of running for WT and aKQ mice in light:dark conditions. The bar graph shows the 7 day average daily running of the WT and aKQ mice in light:dark conditions. There is no significant difference between WT and aKQ mice [t(7,7) = 0.84, *p* = 0.41] (n = 8, 8). Data are presented as mean ± SEM, *p < 0.05 by two‐tailed Student's t‐test.

Systemic metabolic oscillation is achieved by coordination among metabolic organs, including adipose tissue. Given that PPARγ acetylation affects metabolic responses in adipose tissue and its levels are modulated in a diurnal cycle‐dependent manner, we hypothesized that PPARγ acetylation in adipose tissue might participate in regulating metabolic rhythm. The aKQ mice showed an impaired response to insulin at ZT18 but not at ZT6 or ZT12 (**Figure** **4**a–c). Interestingly, ZT18 was the time point that PPARγ acetylation was at the trough in adipose tissues (Figure 1c; Figure S1b, Supporting Information), showing the maximal difference to aKQ mice. The impact on glucose tolerance in the aKQ mice was minimal at ZT0 but slightly worse at ZT12 (Figure 4d,e). Furthermore, the aKQ mutation did not affect circadian locomotor behavior (Figure 4f,g ). These data demonstrate that PPARγ acetylation status in adipose tissue is involved in determining metabolic rhythms independent of circadian locomotor behavior.

**Figure 5:**
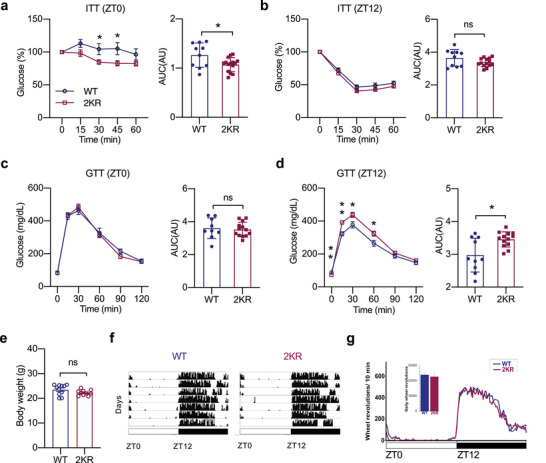
Constitutive deacetylation of PPARγ also alters metabolic rhythm. 13‐weeks‐old male WT or 2KR mice on the chow diet were analyzed at different time points during the diurnal cycle. a,b) ITTs and associated AUC at ZT0 (n = 10, 15) and ZT12 (n = 10, 14); c,d) GTTs and associated AUC at ZT0 (n = 9, 13) and ZT12 (n = 10, 13). e–g) Locomotor activity of 15‐weeks‐old WT and 2KR mice on chow diet feeding (n = 10, 15); e) body weight; f) Actograms show the locomotor activity of representative individual WT and 2KR mice housed in a 12:12 light:dark cycle, as in Figure 4. g) Average daily profile of locomotor activity for WT and 2KR mice in the light dark conditions. The bar graph shows the average daily running in each group [t(7,7) = 0.26, p = 0.80]. Data are presented as mean ± SEM, *p < 0.05 and **p < 0.01 by two‐tailed Students’ t‐test.

If PPARγ acetylation regulates metabolic oscillation during a diurnal cycle, preventing this acetylation should alter the oscillation pattern. In the complementary constitutive deacetylation‐mimetic 2KR mice, there was a significant improvement in insulin sensitivity at ZT0 (**Figure** **5**a); this improvement disappeared at ZT12 (Figure 5b). Moreover, though 2KR mice showed normal glucose tolerance at ZT0 (Figure 5c), they displayed impaired glucose tolerance at ZT12 (Figure 5d). On the other hand, 2KR and WT mice with the same ad libitum body weight displayed no differences in daily wheel running activity (Figure 5e–g). These data complement the findings in aKQ mice that the dynamics of PPAR*γ* acetylation are involved in metabolic rhythm, impacting the oscillations of insulin sensitivity and glucose tolerance.

### 2.6 Adipsin is a Potential Mediator Between PPARγ Acetylation and Metabolic Rhythm

**Figure 6:**
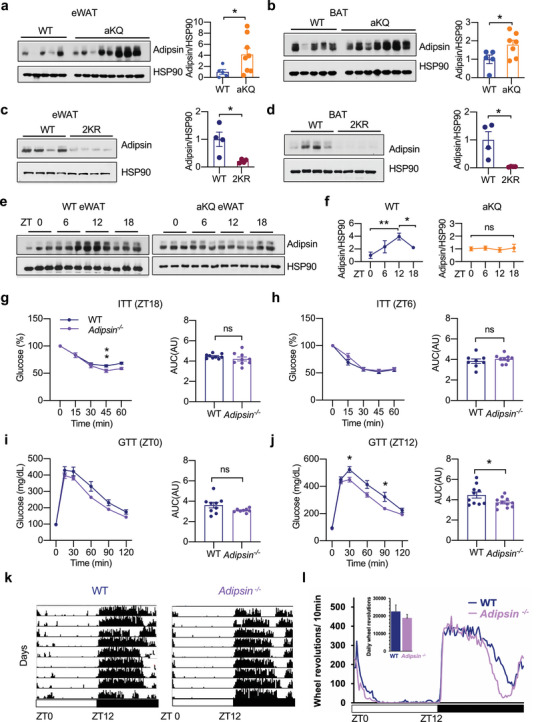
Adipsin is a potential mediator of PPARγ acetylation to affect metabolic rhythm. a,b) WB and quantification of adipsin protein in the eWAT and BAT of HFD‐fed WT and aKQ mice (*n* = 5, 7). c,d) WB and quantification of adipsin protein in the eWAT and BAT of chow‐fed WT and 2KR mice. e,f) WB analysis of adipsin oscillation over a 24 hr time period in the eWAT of chow‐fed WT and aKQ mice and quantification (*n* = 3, 3). g) Metabolic characterizations of 13‐weeks‐old WT and *Adipsin* ^−/−^ mice on chow diet feeding; g,h) ITTs and associated AUC at ZT18 (*n* = 9, 9) and ZT6 (*n* = 8, 9); i,j) GTTs and associated AUC at ZT0 (*n* = 9, 8) and ZT12 (*n* = 10, 11). k,l) Locomotor activity of 15‐weeks‐old WT and *Adipsin* ^−/−^ mice on chow diet feeding (*n* = 11, 11); k) Actograms show the locomotor activity of representative individual WT and *Adipsin* ^−/−^ mice housed in a 12:12 light:dark cycle, as in Figure 4. l) Average daily profile of running for WT and *Adipsin* ^−/−^ mice in light:dark conditions. The bar graph shows the average daily [*t*_(7,7)_ = 0.14, *p* = 0.89]. Data are presented as mean ± SEM, ^*^ *p* < 0.05 and ^**^ *p* < 0.01 by two‐tailed Student's *t*‐test.

PPARγ deacetylation selectively regulates downstream targets, among which is adipsin,^[^ ^19^, ^20^ ^]^ a representative adipokine that correlates with obesity, aging, and type 2 diabetes in humans.^[^ ^30^ ^]^ We observed an increase of adipsin proteins in both the eWAT and BAT of DIO aKQ mice (**Figure** **6**a,b). Consistently, adipsin levels were suppressed in 2KR mice (Figure 6c,d). Despite the extensive studies on adipsin, it has never been linked to diurnal rhythms. Interestingly, we found a rhythmic pattern of adipsin expression in the adipose tissue of lean WT mice, with a peak at ZT12 and a trough at ZT0 (Figure 6e,f). This rhythm was lost in aKQ mice. In contrast, the fluctuation of adipsin was not observed in the plasma (FigureS6a,b,SSupporting Information). These results imply an adipose‐autonomous role of adipsin in metabolic rhythms.

Next, we used adipsin whole‐body knockout mice (*Adipsin* ^*−/−*^) to test its impact on metabolic rhythms directly. *Adipsin* ^−/−^ mice are known to display a minimal metabolic phenotype without metabolic challenges.^[^ ^31^ ^]^ However, with similar body weight as WT mice on a chow diet (Figure S6c, Supporting Information), they displayed a modest improvement in insulin sensitivity at ZT18 (Figure 6g), which was diminished at ZT6 (Figure 6h) and ZT0 (Figure S6d, Supporting Information). Furthermore, glucose tolerance was relatively improved in the *Adipsin* ^−/−^ mice at ZT12 but not at ZT0 (Figure 6i,j). Similar to 2KR and aKQ mice, *Adipsin* ^−/−^ mice showed unchanged locomotor rhythms (Figure 6k,l). Therefore, adipsin exhibits a hitherto unknown function in regulating metabolic oscillation during the rhythm cycle, serving as a potential mediator of PPARγ acetylation.

### 2.7 Adipsin Destabilizes BMAL1 in an Adipocyte‐Intrinsic Manner

**Figure 7:**
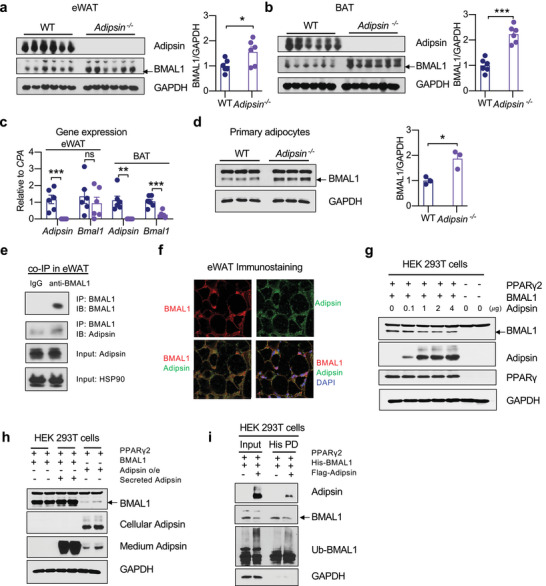
Adipsin destabilizes BMAL1 in adipocytes. a,b) WB and quantification of BMAL1 in the eWAT and BAT of WT and *Adipsin* *^−/−^* mice. c) *Bmal1* gene expression by qPCR analysis (n = 6, 6). d) WB and quantification of BMAL1 protein in WT and *Adipsin* *^−/−^* primary adipocytes (n = 3, 3). e) Co‐immunoprecipitation (IP) of endogenous BMAL1 and adipsin in eWAT. f) Co‐localization of BMAL1 and adipsin in eWAT by immunostaining. Scale bar, 10 𝜇m. g) WB analyzing the dose‐dependent effect of adipsin on BMAL1 stability in HEK 293T cells in the presence of ectopic PPAR*γ*2. h) WB of BMAL1 in HEK 293T cells treated with conditioned medium containing secreted adipsin. i) His‐tagged BMAL1 was pulled down (PD) to examine BMAL1 ubiquitination in HEK 293T cells with adipsin and PPAR*γ*2 co‐overexpression. Data are presented as mean ± SEM, *p < 0.05, **p < 0.01, and ***p < 0.001 by two‐tailed Student's t‐test.

Since PPARγ acetylation destabilizes the master circadian regulator BMAL1 and adipsin is uncovered as a downstream diurnal factor, we asked whether adipsin executes the destabilization of BMAL1. In both eWAT and BAT of *Adipsin* ^−/−^ mice, BMAL1 was augmented (**Figure** **7**a,b). Consistently, the rhythmic pattern of BMAL1 was altered in *Adipsin* ^−/−^ mice and aKQ mice (Figure S7a,b, Supporting Information). Importantly, *Bmal1* expression was not increased and even repressed in the BAT of *Adipsin* ^−/−^ mice (Figure 7c), further emphasizing regulation of BMAL1 at the level of protein stability. To determine whether the increase of BMAL1 in adipose tissue is an adipocyte‐autonomous effect, we compared BMAL1 levels in primary adipocytes derived from WT and *Adipsin* ^−/−^ mice. Despite unchanged adipogenesis,^[^ ^20^ ^]^ ablation of adipsin resulted in a higher level of BMAL1 protein (Figure 7d). Thus, these data imply a novel function of adipsin to destabilize the circadian factor BMAL1.

As adipsin is a secretory protein, we hypothesized that its actions may be felt in other tissues. However, in contrast to the increase of BMAL1 in the WAT and BAT of *Adipsin* ^−/−^ mice, there were comparable levels of BMAL1 in the liver (Figure S7c, Supporting Information). This suggests that adipsin effects on adipose BMAL1 may be local, i.e., non‐hormonal. Indeed, immunoprecipitating BMAL1 in eWAT was able to pulldown endogenous adipsin (Figure 7e), further supported by their colocalization (Figure 7f). This interaction and colocalization were confirmed in vitro in HEK 293T cells (Figure S7d,e, Supporting Information) and in cultured adipocytes (Figure S7f, Supporting Information). Furthermore, adipsin dose‐dependently degraded BMAL1 in HEK 293T cells when PPAR*γ* was co‐overexpressed (Figure 7g), while secreted adipsin failed to decrease BMAL1 (Figure 7h). In the presence of PPARγ, adipsin increased the ubiquitination of BMAL1, underlying its degradative effect (Figure 7i). The BMAL1‐proteolytic function of adipsin appeared to depart from its conventional role to regulate complement activation, as ablation of the adipsin substrate, complement factor C3, did not affect BMAL1 levels in adipose tissue (Figure S7g, Supporting Information). Adipsin also degraded BMAL1 in the presence of a C3aR inhibitor in HEK 293T cells (Figure S7h, Supporting Information). Altogether, these findings reveal a new function of adipsin to mediate the diurnal cycle of adipose metabolism by destabilizing BMAL1.

## 3 Discussion

**Figure 8:**
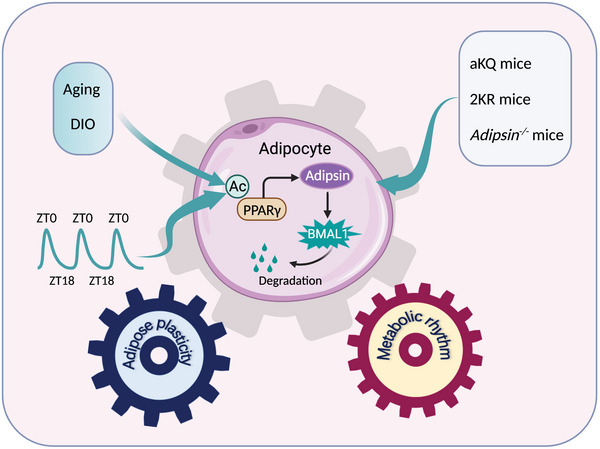
Schematic model of PPARγ acetylation/adipsin pathway through degrading BMAL1 to connect adipose plasticity and metabolic rhythm. The illustration was created with BioRender.

In this study, we addressed the orchestration of metabolic oscillation during the diurnal cycle through the perspective of PPARγ acetylation. PPARγ acetylation in adipose tissue dynamically fluctuates in physiological diurnal rhythms and increases in pathological conditions of obesity, aging, and disruption of the circadian cycle. As a PPARγ acetylation‐responsive downstream target, adipsin is uncovered as a novel diurnal protein and exhibits an unexpected function in destabilizing BMAL1. As such, the dynamics of PPARγ acetylation in adipocytes orchestrate adipose plasticity and metabolic rhythm (**Figure** **8**).

PPARγ acetylation levels are determined by the balance between the enzymatic activities of acetyltransferase and deacetylase, as well as ligand availability. PPARγ is acetylated by CBP/p300 using acetyl‐CoA as the acyl donor.^[^ ^18^ ^]^ In contrast, the PPARγ deacetylase SirT1 is activated in response to NAD^+^. Both acetyl‐CoA and NAD^+^ are involved in energy metabolism.^[^ ^32^ ^]^ PPARγ acetylation is thus likely to be sensitive to metabolic status, particularly given the vital functions of PPARγ in glucose and lipid metabolism. Moreover, declined NAD+ levels have been associated with aging, obesity, and type 2 diabetes,^[^ ^33^ ^]^ and supplementing NAD precursor shows improvements in energy metabolism, diurnal rhythm, and glucose metabolism in obesity and aging.^[^ ^34^ ^]^ These facts not only provide an explanation of the increased PPARγ acetylation in metabolic disturbances of aging, obesity, and shiftwork but also raise the possibility of harnessing PPARγ acetylation via manipulating NAD+ levels to tackle metabolic dysfunctions. This possibility is further supported by a catabolic phenotype in the constitutive deacetylation‐mimetic 2KR mice ^[^ ^19^ ^]^ and the consistent decrease of energy expenditure in aKQ mice. Hence, the acetylation status of PPARγ is closely involved in metabolic adaption.

aKQ mice lost plasticity in fat accumulation in response to HFD feeding and fat reduction during CR, likely through different mechanisms. The inhibited fat reduction in CR is in line with their lower efficiency in energy expenditure, whereas the inhibited fat accumulation on HFD feeding is underlain by impaired adipogenesis. Adipogenesis is fundamental to healthy adipose tissue expansion in response to calorie challenge.^[^ ^35^ ^]^ Interestingly, this anti‐obesity phenotype is age‐dependent and disappears in elder aKQ mice. There are two possible explanations: (i) Younger mice undergo active adipogenesis to increase fat mass, whereas in old age, adipogenesis slows down, and the increase in fat mass is mainly supported by adipocyte hypertrophy. (ii) Acetylation of endogenous WT PPARγ increases during aging. There is likely a stronger contrast regarding the acetylation status of aKQ mutant to WT PPARγ at a younger age, and this difference is diminished with age. Nevertheless, the KQ mutation inhibited adipogenesis in primary adipocytes and engineered PPARγ reconstitution cell lines. This inhibition of adipogenesis by PPARγ acetylation is consistent with the function of deacetylase SirT1, which is temporarily induced in early adipogenesis.^[^ ^36^ ^]^

Considering the fluctuations of PPARγ acetylation during the diurnal cycle, it is not surprising to observe the diurnal expression of its downstream target adipsin, which is disrupted in aKQ mice. These data are the first demonstration of adipsin as a diurnal factor. Of note, we observe a clear peak shift between PPARγ acetylation and adipsin expression. In eWAT, the former peaked at ZT0 and reached a trough at ZT18. In contrast, the abundance of adipsin increased from ZT0 and reached a peak at ZT12. This difference in timing is probably caused by the interval between PPARγ acetylation‐dependent gene transcription and accumulation of the translated protein product in adipose tissue, as well as loss to plasma. Given the positive association of plasma adipsin levels with obesity, aging, and type 2 diabetes in humans,^[^ ^30^ ^]^ our findings highlight that the loss of adipsin diurnal regulation might be a contributing risk factor.

This study suggests adipsin as the link between PPARγ acetylation and daily rhythms via destabilization of BMAL1. In the classic view, adipsin is a cytokine secreted by adipocytes to promote C3 cleavage and activate the alternative complement system.^[^ ^37^ ^]^ The novel function of adipsin in destabilizing BMAL1 departs from this established dogma in several ways. First, it is a cell‐intrinsic phenomenon that does not require endocrine regulation, as the upregulation of BMAL1 in *Adipsin* ^−/−^ mice was only observed in adipose but not distant tissues, nor did secrete adipsin degrade BMAL1. Second, knockout of C3 did not increase BMAL1 in adipose tissue, indicating an independent mechanism from the traditional role of adipsin to regulate complement activity. Third, adipsin was found to colocalize and interaction with BMAL1. Overexpressing adipsin in HEK 293T cells induced BMAL1 degradation when PPARγ*γ*was co‐overexpressed. This further suggests an adipocyte‐specific regulatory pattern, in which both adipsin and PPARγ are present in high abundance. We did not detect an interaction between adipsin and PPARγ, suggesting that the degradation of BMAL1 by adipsin may require PPARγ to activate some downstream targets to facilitate BMAL1 degradation by adipsin. Despite the fact that the regulation of metabolism by circadian clocks has been extensively studied,^[^ ^38^ ^]^ the destabilization of BMAL1 by adipsin highlights a novel potential mechanism of deregulation of circadian clocks in aging and obesity^[^ ^13^, ^39^ ^]^ and how this may connect with metabolic dysfunction.

## 4 Conclusions

In summary, our findings demonstrate that PPARγ acetylation in adipose tissue dynamically connects adipose plasticity and metabolic rhythm. Furthermore, we identified adipsin as a potential mediator and novel diurnal factor that destabilizes BMAL1. By uncovering the role of PPARγ acetylation in regulating metabolic rhythms, our study presents the possibility that approaches to manipulate PPARγ acetylation in a circadian‐dependent manner may restore healthy metabolic oscillations in obesity and aging.

## 5 Experimental Section

### Animals Studies

The aKQ and 2KR mouse lines were bred and maintained on the C57BL/6 (Stock No: 000664) background. The 2KR and *Adipsin* ^−/−^ mouse lines were as described previously.^[^ ^19^, ^20^ ^]^ The Adipoq‐Cre mouse line (Stock No: 02 8020) was purchased from Jackson Laboratory (Maine, USA). For the shiftwork study, male C57BL/6 mice were purchased from the Jackson Laboratory (Maine, USA). To continuously measure diurnal activity, animals were individually housed in translucent propylene cages (29 × 19 × 12.5 cm) equipped with a running wheel (11 cm diameter), and water and chow were provided ad libitum. Uniform illumination of cages was provided by an array of green LEDs (230 lux, peak wavelength 524 nm, half‐maximal width 47 nm, mean dominant wavelength 518 nm, HLMP‐AM01‐Q00zz; Avago Technologies, San Jose, CA, USA). Environmental noise was masked by white noise (76 dB SPL). Animals were adapted to their home cages and to the 12:12 light:dark (LD) cycle for 2 weeks before the start of each experiment.

The mice were regularly fed on a chow diet (Purina 5053, 24.7% kcal from protein, 62.1% carbohydrate, and 13.2% fat). The HFD contains 60% calories from fat, 20% from protein, and 20% from carbohydrates (Research Diets: D12492i). In the CR experiment, mice were given 70% of their daily food intake (2.8 g per mouse per day). All experiments were reviewed and approved by the Columbia University Animal Care and Utilization Committee (IACUC).

For the shiftwork study, mice at the age of 12 weeks were fed ad libitum and grouped‐housed in a 12:12 LD cycle to acclimate for 2 weeks. To assess the effects of shiftwork like phase changes, half of the cohort underwent phase advance shifts of 6 h once per week four times, thereby returning to the initial lighting condition for 2 weeks before they were sacrificed. The control animals were maintained at 12:12 LD for the duration of the study. Phase‐shifted and control mice were sacrificed ad libitum feeding.

The metabolic cage experiment was performed using the Comprehensive Lab Animal Monitoring System (CLAMS) (Columbus Instruments, Columbus, Ohio, USA) at ambient temperature (RT, 23 ± 1 °C) on a 12 h light (7 am) /12 h dark cycle (7 pm) with access to food and water ad libitum.

### Locomotor Activity

Wheel running data were collected continuously in 10 min bins using VitalView (Starr Life Science Corp, Oakmont, PA.) and were quantified by Actiview (Starr Life Science). Activity profiles were generated in Microsoft Excel. Daily activity was established by measuring total wheel revolutions for 24 h.

### Metabolic Tests

For the insulin tolerance test (ITT), mice were fasted for 5 h in cages with fresh bedding and intraperitoneally (i.p.) injected with insulin (0.75 U insulin kg^−1^ BW). Blood glucose was measured by ONETOUCH Ultra glucometer at 0, 15, 30, 45, and 60 min after injection. For the glucose tolerance test (GTT), mice were fasted for 16 h, except for a 24 h fast for the 7 pm test. After fasting, mice were injected with glucose (i.p., 2 g kg^−1^ BW). Blood glucose was measured at 0, 15, 30, 60, 90, and 120 min after injection. Body compositions were examined using EchoMRI (EchoMRI LLC, Houston, TX, USA).

### Generation of PPAR*γ* Acetylation‐Mimetic aKQ Mice

The Lysine 293 site (AAA) on the exon 6 of Pparg was mutated to Glutamine (CAG) (Figure S2a, Supporting Information), termed KQ mutant. The targeting approach is outlined in Figure S2b (Supporting Information). The linearized targeting vector was transfected into mouse embryonic stem (ES) cells for recombination, and G418‐resistant clones were screened by Long Range PCR and Southern blotting and further confirmed by genomic DNA sequencing. Blastocyst injection was conducted to obtain chimeric mice on a C57BL/6 background, which were bred with WT C57BL/6 mice to obtain F1 heterozygotes. Heterozygous F1 mice carrying the KQ allele were identified by genotyping and genomic DNA sequencing and then bred with Adipoq‐Cre mice on a C57BL/6 background to obtain *Pparg* ^KQ/KQ^: Adipoq‐Cre mice (aKQ mice), using *Pparg* ^KQ/KQ^ littermates as controls. aKQ homozygote mice were born in the Mendelian ratio.

### Gene Expression Analysis

Total RNA from tissues or cells was isolated using TriZol (Sigma–Aldrich). After mixing with 300 µL chloroform, RNA was extracted using the NucleoSpin RNA Kit (Macherey‐Nagel, Inc). The High‐Capacity cDNA Reverse Transcription Kit (Applied Biosystems) was used to synthesize cDNA from 1 µg total RNA. Quantitative real‐time PCR (qPCR) was completed on a Bio‐Rad CFX96 Real‐Time PCR system using GoTaq qPCR Master Mix (Promega). The relative gene expression levels were calculated using the ΔΔCt method and *Cyclophilin A* *(CPA)* or Ribosomal Protein L23 (Rpl23) as the reference gene.

### Immunoprecipitation

Fat tissue was homogenized in 1 mL of ice‐cold IP buffer (1x TBS, 1% triton, 10% glycerol, 1% protease inhibitor cocktail, 3 µmol trichostatin A, 20 mmol nicotinamide, and 20 mmol sodium butyrate). Protein lysates were incubated on ice for 10 min before sonication for 3—5 min and then centrifuged for 10 min at maximum speed. Extra IP buffer was added to ≈300–500 µg of protein lysate to 1 mL. This solution was then mixed with 30–40 µL anti‐Acetyl‐Lysine (mAb mix) Affinity Beads (Cytoskeleton, Inc., AAC04‐beads), anti‐BMAL1 (Proteintech, #14268‐1‐AP), or Flag M2 beads (Sigma–Aldrich, SLCD9431). The samples were rotated at 4 °C for overnight and spun down at 1,200 rpm for 1 min at 4 °C. The supernatant was discarded, and the beads were washed with 1 mL of IP wash buffer >3 times. 30–60 µL 1x non‐reducing loading buffer (50 mmol Tris pH 6.8, 1% SDS, and 10% Glycerol) was added to the beads and incubated at room temperature for 5 min. The samples were then heated at 95 °C for 5 min to elute the immunoprecipitated proteins. After centrifugation, the supernatant was subjected to SDS‐PAGE for analysis by Western blotting.

### His‐tagged Protein Pulldown

Cells overexpressing 6x His‐tagged BMAL1 (Addgene #31367) were resuspended in 700 µL Binding Buffer (50 mmol Sodium Phosphate pH 8.0, 300 mmol NaCl, 0.01% TweenTM‐20, 1%Triton X‐100). The samples were vortexed and incubated on ice for 10 min, followed by sonication for 4 min and centrifugation at maximum speed for 10 min. The supernatant was collected, and the protein concentration was measured using the Pierce BCA Protein Assay Kit (Thermo Scientific, catalog23227). Fifty microliters of Dynabeads His‐tag beads (Invitrogen, catalog 10103D) were added to the protein lysates and rotated at 4 °C for 10 min. Afterward, the beads were washed 4 times in 300 µL 1x Binding Buffer and eluted in 100 µL His‐Elution Buffer (300 mmol Imidazole, 50 mmol Sodium‐ phosphate pH 8.0, 300 mmol NaCl, 0.01% TweenTM‐20).

### Western Blotting

Cells or tissues were extracted in the IntactProtein Lysis Buffer (GenuIn Biotech #415). SDS‐PAGE separation was performed using 8–10% gels. Western blotting was performed and detected with Pierce ECL Western Blotting Substrate (Thermo Scientific, catalog PI32209). Antibodies used in this study are as follows: anti‐PPAR*γ* (Cell Signaling Technology, #2443), anti‐aP2 (Cell Signaling Technology, #2120), anti‐Perilipin (Cell Signaling Technology, #9349), anti‐ BMAL1 (Proteintech, #14268‐1‐AP, ThermoFisher Scientific, #PA1‐523), anti‐ubiquitin (Proteintech, #10201‐2‐AP), anti‐adipsin (R&D systems, #AF5430), anti‐C3 (Proteintech, #21337‐1‐AP), anti‐GAPDH (Proteintech, #HRP‐60004), anti‐HSP90 (Proteintech, #13171‐1‐AP), anti‐C/EBP𝛼 (Santa Cruz, #sc‐61), anti‐Rabbit IgG‐HRP produced in goat (Sigma, #A0545), anti‐sheep IgG (R&D, # HAF016).

### Cell Culture

To generate the *Pparg^–/–^* reconstituted cell line, the cDNAs of PPAR*γ* variants PPAR*γ*2‐WT and PPAR*γ*2‐KQ were cloned into the pTRIPZ plasmid, which contained a doxycycline‐inducible regulatory element (Thermo Open Biosystems). These constructs were used to generate MEFs that stably overexpressed *Pparg^–/–^* as described in the selection of puromycin (2.5 µg mL^−1^).^[^ ^40^ ^]^ Cells were cultured in high‐glucose DMEM with 10% FBS (Corning) and 1X penicillin/streptomycin (Thermo Fisher). Two days prior to adipocyte induction, cells were treated with 1 µg mL^−1^ doxycycline to induce PPARγ expression. The adipogenic medium included 10 µg mL^−1^ insulin, 1 µmol dexamethasone, and 0.5 mmol 3‐isobutyl‐1‐methylxanthine in the presence or absence of 5 µmol Rosi. After induction for 2 days, cells were changed into the medium containing 1 µg mL^−1^ doxycycline and 2.5 µg mL^−1^ insulin with or without 5 µmol Rosi until mature adipocytes formed. The lipid content was examined using BODIPY staining. C3H10T1/2 cells were purchased from ATCC and cultured in high glucose DMEM supplemented with 10% FBS and 1 X penicillin/streptomycin (Thermo Fisher). After reaching confluence for 2 days, cells were differentiated in the same induction medium as described above in the presence of 5 µmol Rosi for another 2 days. Afterward, cells were maintained in the medium containing 2.5 µg mL^−1^ insulin until fully differentiated. The HEK 293T cell line was cultured in high glucose DMEM supplemented with 10% FBS and 1 X penicillin/streptomycin (Thermo Fisher).

### Primary Adipocyte Culture

Inguinal fat pads with the lymph nodes removed were dissected from WT and aKQ mice at the age of 4–6‐weeks‐old. Stromal Vascular Fraction (SVF) cells were extracted and cultured as described in the previous study.^[^ ^20^ ^]^ The isolated adipose stromal cells (ASCs) were expanded and differentiated into adipocytes using a standard adipogenic induction medium previously outlined in the presence or absence of 5 µmol Rosi for 2 days. Cells were kept in the maintenance medium until fully differentiated.

### Immunostaining

Cultured cells were washed in PBS and fixed in 4% paraformaldehyde for 20 min at room temperature, followed by incubation in membrane permeabilization solution (0.2% Triton‐X in PBS) for 20 min. Cells or tissue sections were blocked in 5% goat serum for 1 h and incubated with anti‐ BMAL1 and anti‐adipsin antibodies overnight at 4 °C. Fluorescent secondary (1:400 dilution) antibodies AF488 and AF647 (Thermo Fisher Scientific) were used. Cells or tissue sections were washed and incubated in 4′,6‐diamidino‐2‐phenylindole (DAPI) (1:1000 dilution) for nuclear staining. Images were taken at 40× magnification on a Zeiss confocal microscope with an LSM 710 scanning module.

### Hematoxylin and Eosin (H&E) Staining

After dissection, eWAT and BAT were immediately fixed in 10% formalin buffered solution overnight and dehydrated in 70% ethanol at 4 °C for 2 days. Tissues were embedded into the paraffin and cut into 5 µm sections and were stained with hematoxylin and eosin (H&E). The images were photographed under a microscope (Olympus I X 71) with a DP74 camera.

### Statistics

Quantitative data are expressed as mean ± SEM (standard error of measurement). Statistical analyses were performed using Prism 6.0 software (GraphPad Software, San Diego, CA, USA). Two‐tailed Student's *t*‐tests were applied for comparisons between the two groups. One‐way ANOVA was used for comparisons among three or more groups, followed by Tukey's multiple comparison test. A *p*‐value <0.05 was regarded to be statistically significant.

## Conflict of Interest

The authors declare no conflict of interest.

## Author Contributions

L.Q. and Y.H. conceptualized the project, formulated the initial study design, and wrote the original draft. Y.H. performed the experiments with help from L.Y., R.Z., and T.Z.. L.L., L.F., N.A., and R.Z. helped with data analysis. R.S. developed the method for circadian studies with support from A.B.T., Y.Y., and J.L. in conducting the experiments. N.A., R.T., J.W., U.P., L.W., and R.S. discussed and edited the manuscript. L.Q. acquired funding and supervised the project. All authors approved the final manuscript.

## Supporting information

Supporting Information

## Data Availability

The data that support the findings of this study are available from the corresponding author upon reasonable request.
