# Supplementary material for: PPARγ Acetylation Orchestrates Adipose Plasticity and Metabolic Rhythms
Source: Adv Sci (Weinh). 2022 Nov 17;10(2):2204190. doi: 10.1002/advs.202204190 (PMC9839851; doi:10.1002/advs.202204190)
Supplement: Supplementary file 1 — Supporting Information [file ADVS-10-2204190-s001.pdf]

ADVANCED SCIENCE
Open Access

Supporting Information

for *Adv. Sci.*, DOI 10.1002/advs.202204190

PPARγ Acetylation Orchestrates Adipose Plasticity and Metabolic Rhythms

*Ying He, Alana B'nai Taub, Lexiang Yu, Yifan Yao, Ruotong Zhang, Tarik Zahr, Nicole Aaron, Joseph LeSauter, Lihong Fan, Longhua Liu, Ruya Tazebay, Jianwen Que, Utpal Pajvani, Liheng Wang, Rae Silver and Li Qiang**

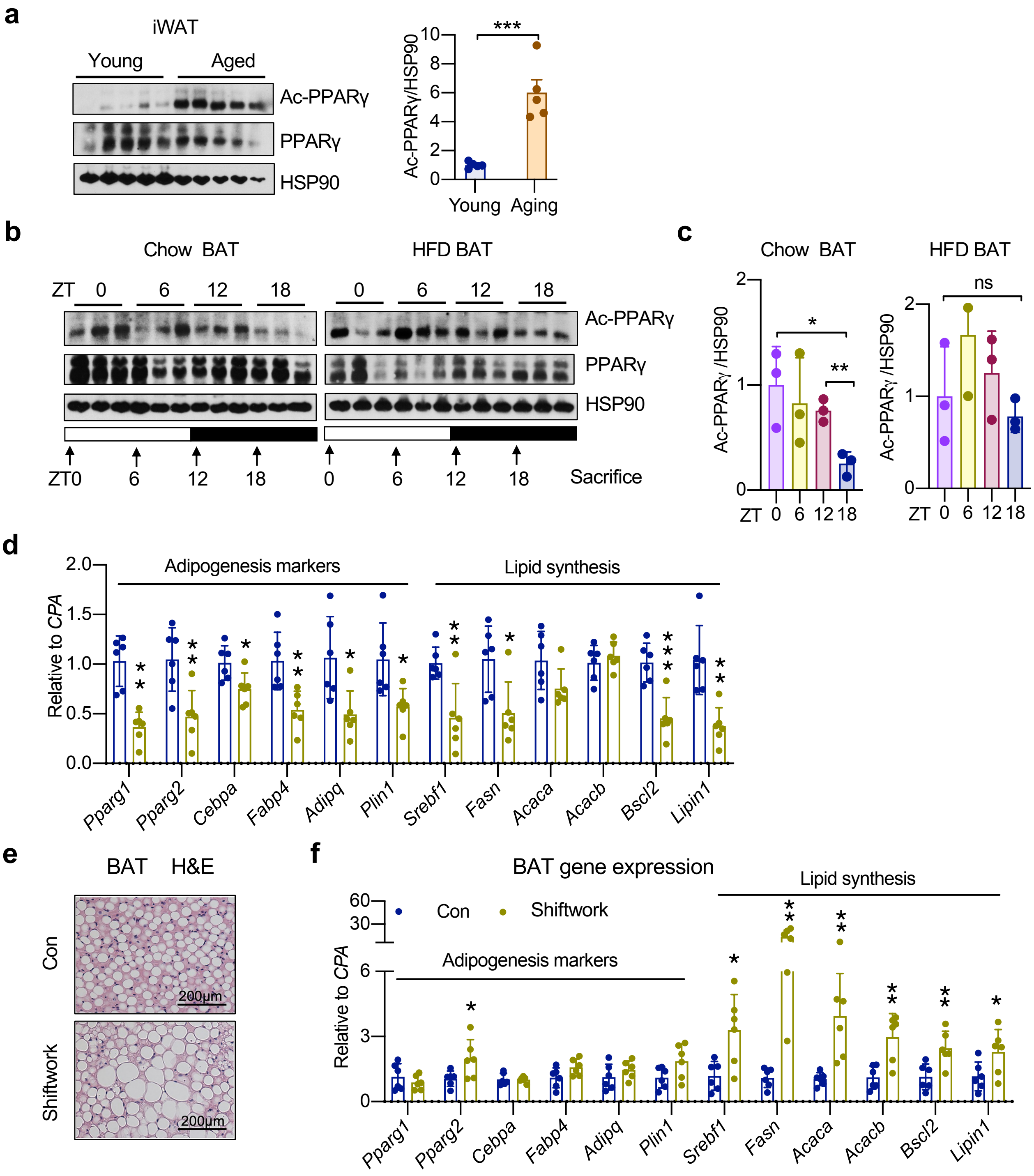

Figure S1. PPARγ acetylation levels in fat are associated with metabolic conditions. a) WB analysis and quantification of PPARγ acetylation levels in the iWAT of young (8-wk-old) and aging (40-wk-old) male mice. b,c) WB analysis of PPARγ acetylation levels in BAT of chow-fed and DIO mice sacrificed at 6-hour intervals throughout one light:dark cycle (b) and the quantification (n = 3) (c). d-f) Analyses in the regular (control) and shiftwork mice (n = 6, 6): d) qPCR analysis of adipogenic and lipogenic genes in the eWAT; e) the whitening morphology of BAT in the shiftwork mice by Hematoxylin and Eosin (H&E) staining; f) Upregulation of lipogenic genes in the BAT of shiftwork mice. Data are presented as mean ± SEM, *P < 0.05, **P < 0.01, ***P < 0.001 by two-tailed Student's t-test.

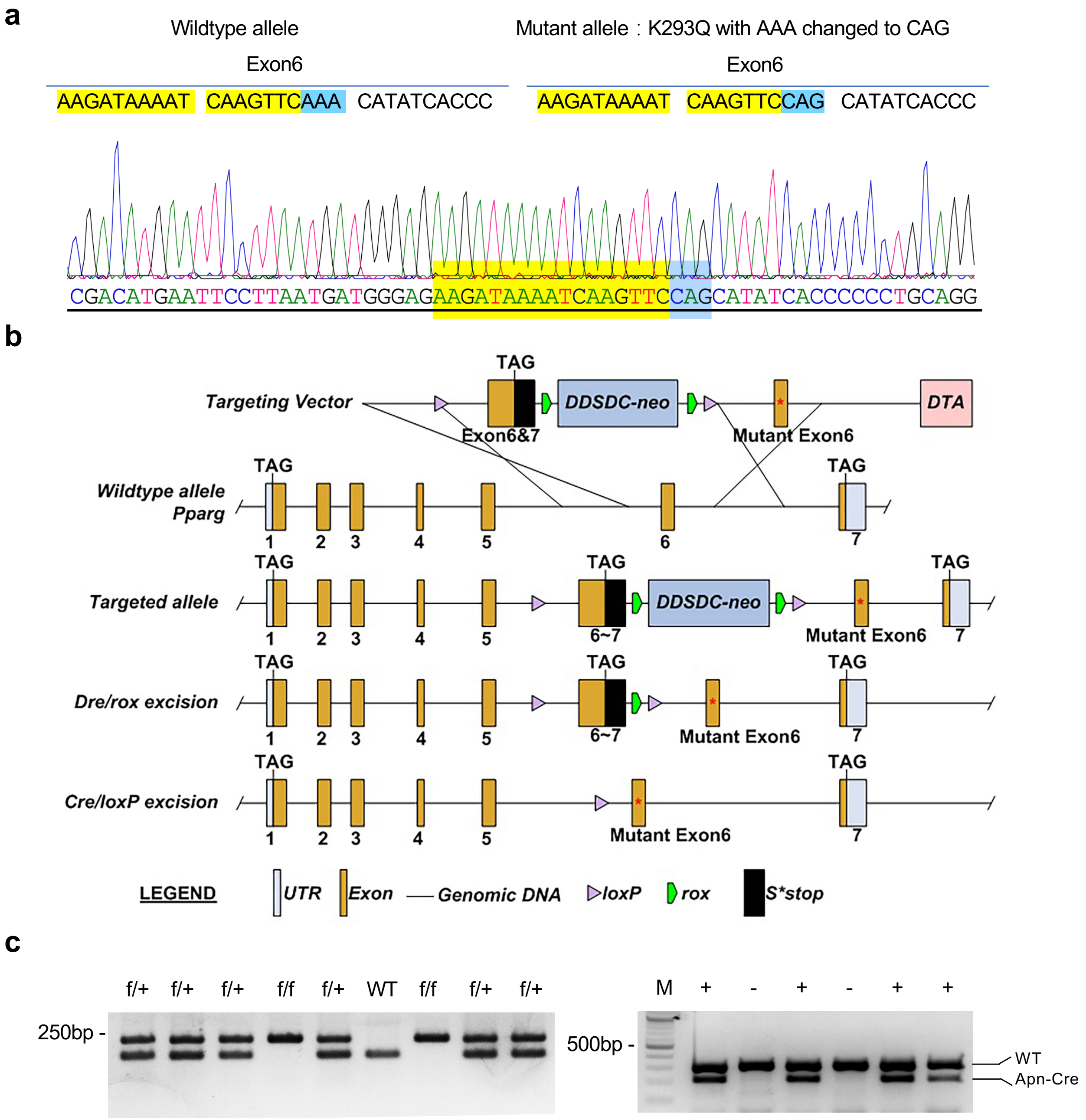

Figure S2. Generation of acetylation-mimetic aKQ knock-in mouse model. a) PPARγ cDNA sequencing of cloned from adipose tissue confirming the replacement of PPARγ WT allele with the K293Q allele. The mutated nucleotides are highlighted in blue. b) Overview of the targeting strategy for generating conditional KQ floxed mice. c) Genotyping results of the KQ loxP and *Aidipoq*-Cre alleles.

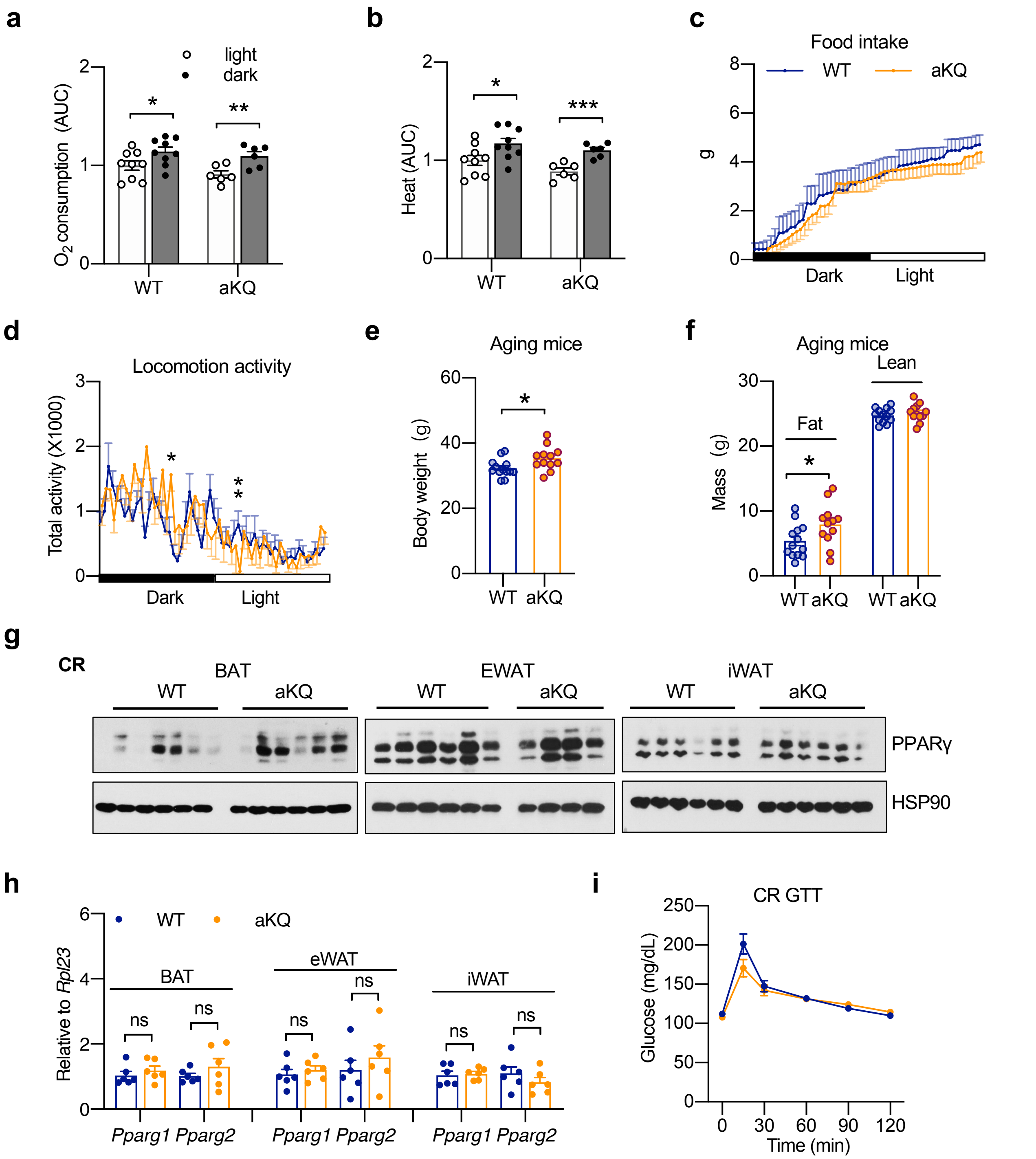

Figure S3. Metabolic characterizations of chow-fed aKQ mice. a-d) Indirect calorimetric analyses of 14-wk-old WT and aKQ mice fed a chow diet at ambient temperature (n = 9, 6); a) Oxygen consumption area under curve (AUC) in light and dark phases; b) heat production AUC in light and dark phases; c) accumulative food intake in one dark:light cycle; d) locomotor activity; *P < 0.05 at indicated detection points; e,f) In 1-year-old male WT and aKQ mice fed a chow diet (n = 13,13): e) body weight; f) body composition. g-i) In male WT and aKQ mice subjected to calorie restriction (CR) for 4 weeks (n = 6, 6): g) WB analysis of PPARγ protein levels in adipose tissues; h) qPCR analyses of *Pparg* expression; i) GTT at 3 weeks of CR. Data are presented as mean ± SEM, *P < 0.05, **P < 0.01, n.s.: not significant by two-tailed Student's *t*-test.

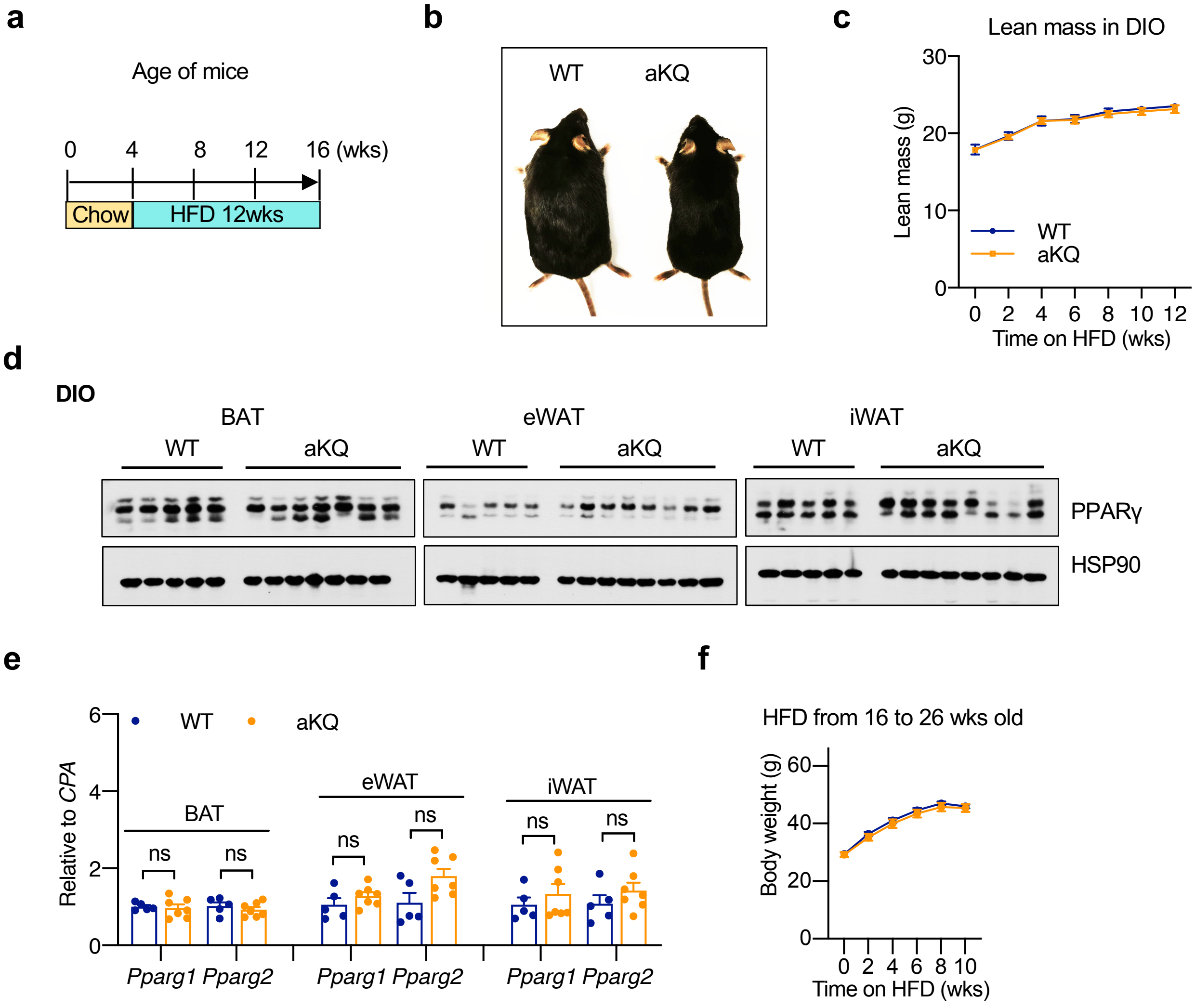

Figure S4. The obesity-resistant phenotype in aKQ mice is age-dependent. a-e) Male control and aKQ mice started on HFD feeding at 4 weeks old (n=5, 8): a) schematic experimental design; b) representative mouse pictures after 12 weeks HFD feeding; c) lean mass curve on HFD feeding; d) WB analysis of PPARγ protein levels in adipose tissues; e) qPCR analyses of *Pparg* expression. f) Body weight curve of male WT and aKQ mice fed HFD from 16 weeks old for 10 weeks (n=13, 9). Data are presented as mean ± SEM, **P* < 0.05, ***P* < 0.01, n.s.: not significant by two-tailed Student's *t*-test.

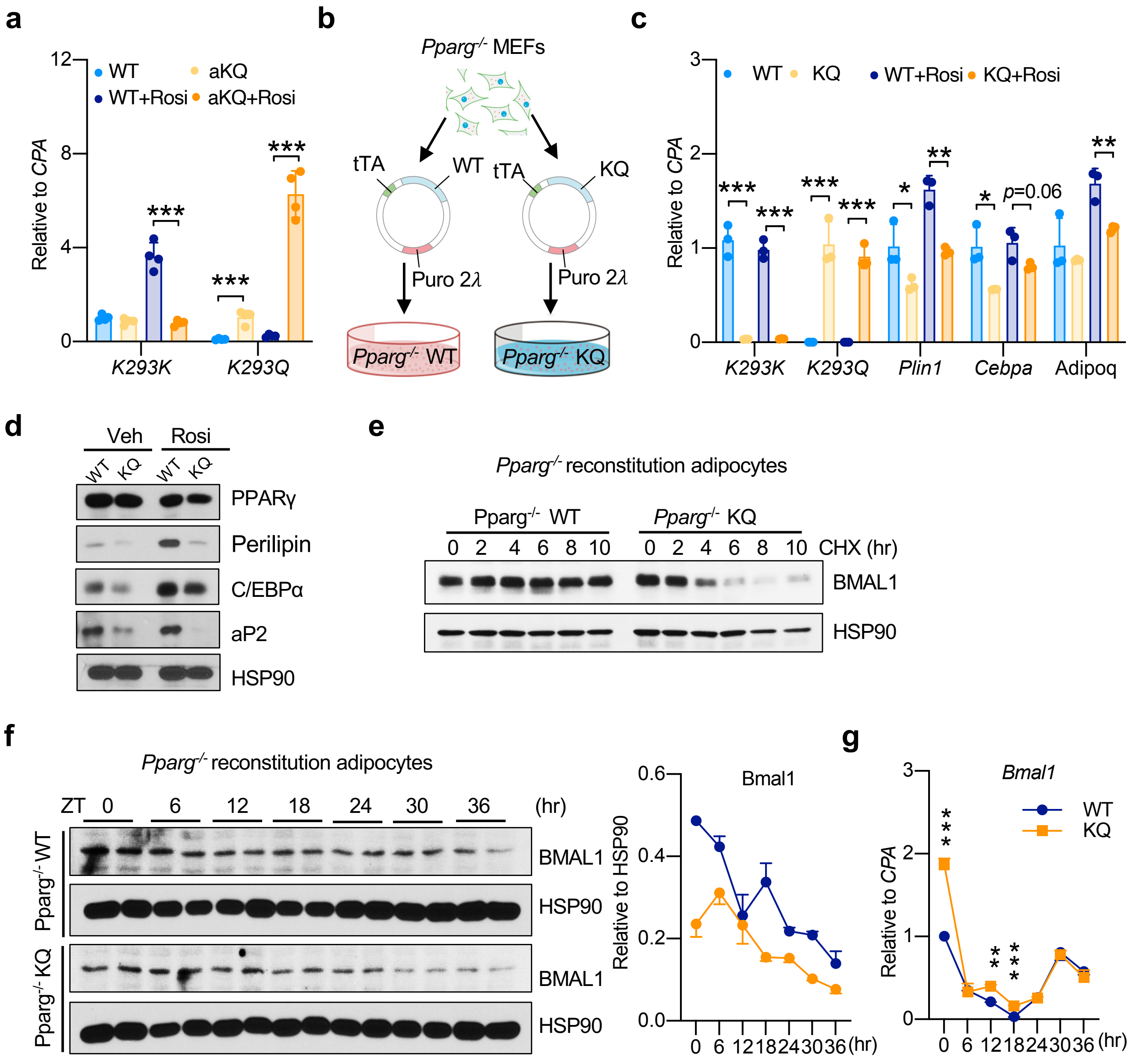

Figure S5. PPARγ acetylation inhibits adipogenesis and destabilizes BMAL1. a) qPCR validation of the KQ mutation in primary adipocytes differentiated with or without Rosi treatment (*n* = 4, 4); b) Schematic diagram of generating the *Pparg*-/- MEF stable cell lines with reconstituted WT or KQ PPARγ created using BioRender. c,d) *Pparg*-/- MEFs reconstituted with WT or KQ PPARγ were differentiated into adipocytes with or without Rosi treatment (*n* = 3, 3): c) gene expression of Pparg WT and KQ mutant and adipogenic markers; d) WB of adipogenic markers. e) BMAL1 protein stability in WT and KQ cell lines during cycloheximide (CHX) treatment time course. f,g) Differentiated adipocytes treated with horse serum treatment for 2 hours to synchronize circadian proteins and then harvested over a 36-hr time course: f) WB analysis of BMAL1 oscillation and quantification (*n* = 2, 2); g) BMAL1 gene expression oscillation (*n* = 3, 3). Data are presented as mean ± SEM, **P* < 0.05, ***P* < 0.01, ****P* < 0.001 by two-tailed Student's *t*-test.

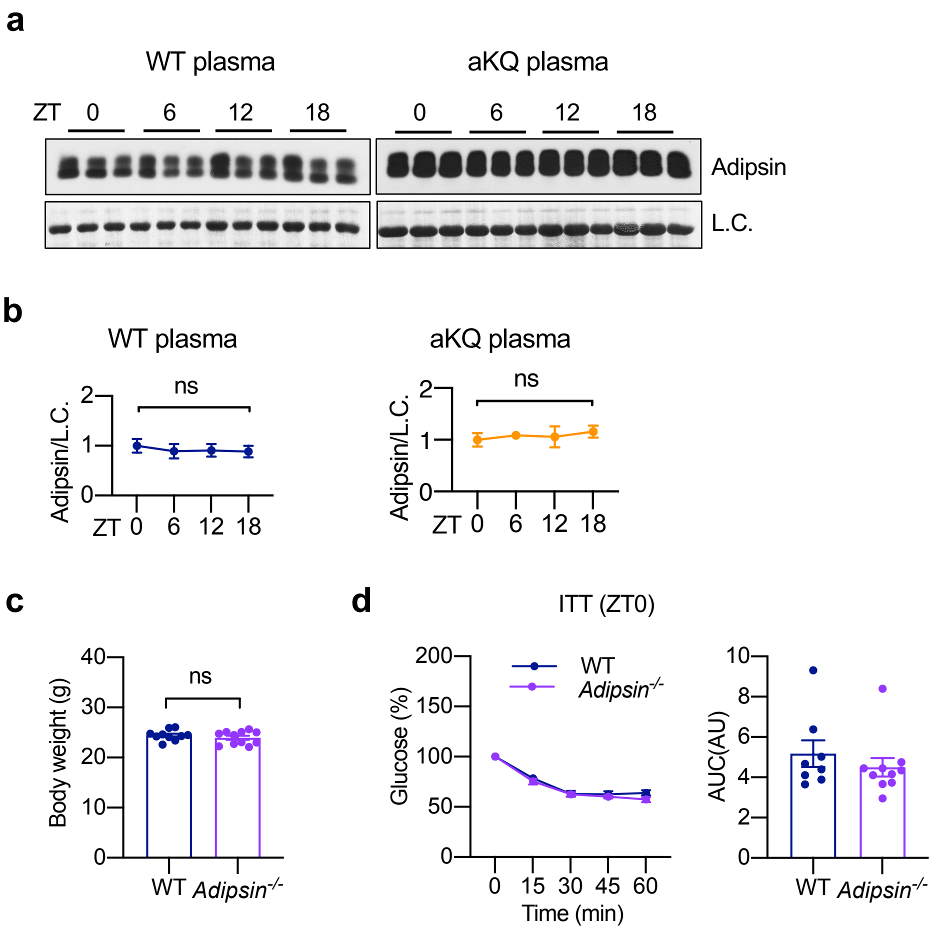

Figure S6. BMAL1 is not destabilized by adipsin in a hormonal manner. a,b) WB and quantification of adipsin protein in the plasma of 19-wk-old WT and aKQ mice on chow diet feeding. Coomassie blue staining as loading control (L.C.). c,d) In the WT and *Adipsin*$^{-/-}$ mice on chow diet feeding, c) no difference in body weight (n = 10, 11); d) ITT and AUC at ZT0 (n = 8, 9). Data are presented as mean ± SEM, n.s. not significant by two-tailed Student's t-test.

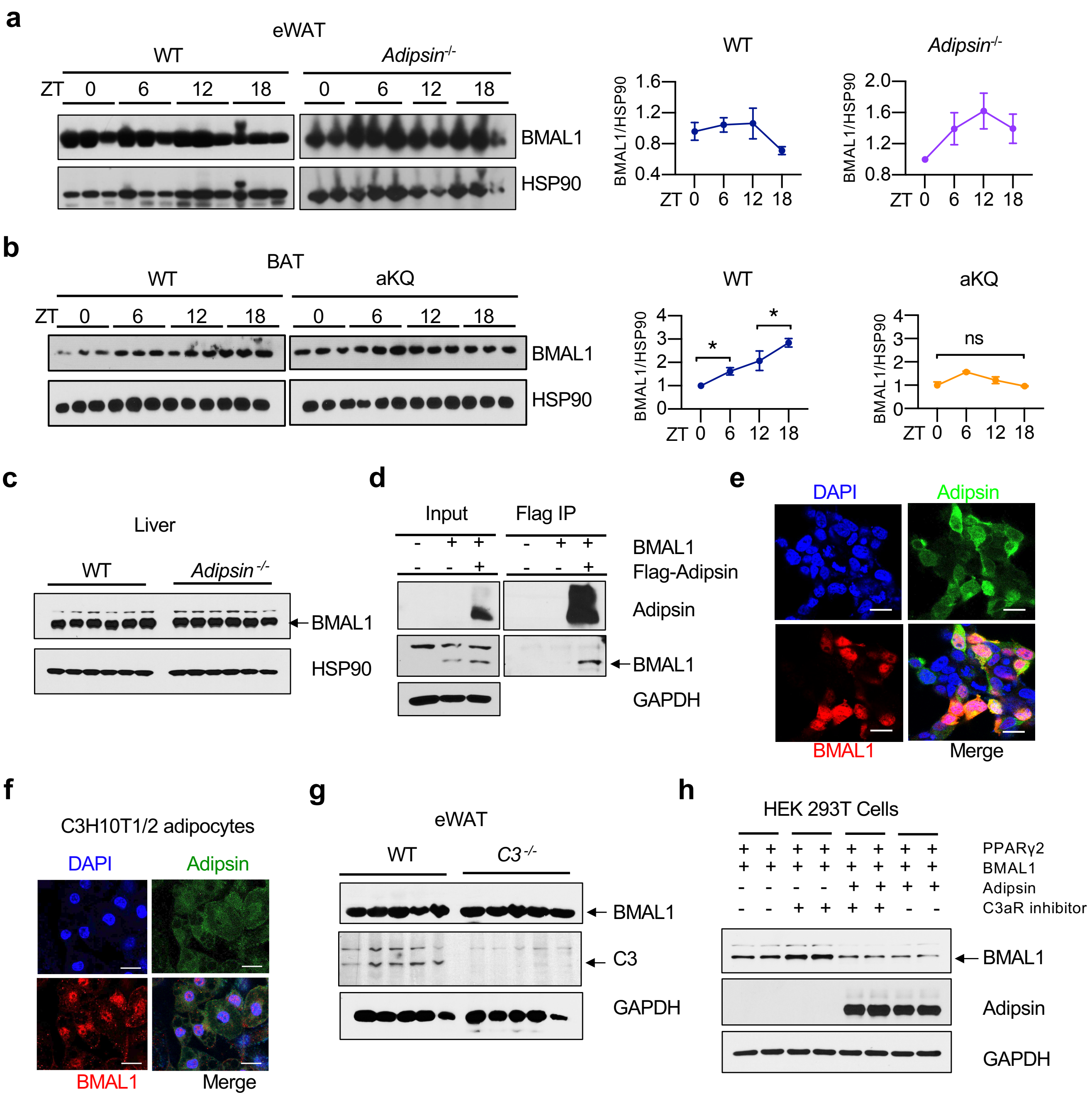

Figure S7. Adipsin regulates BMAL1 expression rhythm through direction a noncanonical manner. a) WB analysis of BMAL1 oscillation over a 24-hour time period in the eWAT of chow-fed WT and *Adipsin*$^{-/-}$ mice and quantification (*n* = 2 or 3). b) WB analysis of BMAL1 oscillation over a 24-hour time period in the BAT of chow-fed WT and aKQ mice and quantification (*n* = 3). c) WB of BMAL1 protein in the liver of WT and *Adipsin*$^{-/-}$ mice on chow diet feeding. d) Co-immunoprecipitation (IP) of Flag-tagged adipsin and BMAL1 in HEK 293T cells. e) Co-localization of overexpressed BMAL1 and adipsin in HEK 293T cells by immunostaining. Scale bar, 20 μm. (F) Co-localization of endogenous BMAL1 and Adipsin in C3H10 T 1/2 adipocytes by immunostaining. Scale bar, 20 μm. g) WB of BMAL1 protein in the eWAT of DIO WT and *C3*$^{-/-}$ mice. h) WB of BMAL1 protein in HEK 293T cells with or without 1 μmol C3aR inhibitor SB290157 treatment for 24 hr. Data are presented as mean ± SEM, n.s. not significant by two-tailed Student's *t*-test.
